# Liquid biopsy of extracellular vesicle biomarkers for prostate cancer personalized treatment decision

**DOI:** 10.20517/evcna.2021.20

**Published:** 2022-01-13

**Authors:** Meng Han, Bairen Pang, Cheng Zhou, Xin Li, Qi Wang, Junhui Jiang, Yong Li

**Affiliations:** ^1^Translational Research Laboratory for Urology, the Key Laboratory of Ningbo City, Ningbo First Hospital, The Affiliated Hospital of Ningbo University, Ningbo 315600, Zhejiang, China.; ^2^Ningbo Clinical Research Center for Urological Disease, Ningbo 315600, Zhejiang, China.; ^3^St George and Sutherland Clinical School, Faculty of Medicine, UNSW Sydney, Kensington, NSW 2052, Australia.; ^4^Cancer Care Centre, St. George Hospital, Kogarah, NSW 2217, Australia.

**Keywords:** Prostate cancer, liquid biopsy, extracellular vesicle, biomarker, early diagnosis, risk prediction

## Abstract

Liquid biopsy of tumor-derived extracellular vesicles (EVs) has great potential as a biomarker source for prostate cancer (CaP) early diagnosis and predicting the stages of cancer. The contents of EVs play an important role in intercellular communication and have specific expression in blood and urine samples from CaP patients. Powered by high-throughput, next-generation sequencing and proteomic technologies, novel EV biomarkers are easily detected in a non-invasive manner in different stages of CaP patients. These identified potential biomarkers can be further validated with a large sample size, machine learning model, and other different methods to improve the sensitivity and specificity of CaP diagnosis. The EV-based liquid biopsy is a novel and less-invasive alternative to surgical biopsies which would enable clinicians to potentially discover a whole picture of tumor through a simple blood or urine sample. In summary, this approach holds promise for developing personalized medicine to guide treatment decisions precisely for CaP patients.

Prostate cancer (CaP) is one of the most common malignancies in male in Western countries. With economic development and lifestyle changes, the incidence and mortality rate of CaP among Asian males have been rising rapidly^[1,2]^. In China, CaP is the leading male genitourinary malignant tumor, and its incidence is now higher than bladder cancer^[3]^. In 2020, CaP caused more than 50,000 deaths in China, which were nearly twice as high as in the United States^[4]^. The survival of CaP patients is highly dependent on tumor stage and risk classification. It was reported that low-risk and localized CaP achieved a favorable prognosis by long-term close monitoring, while metastatic patients had a median survival of 30 months^[5]^. The treatment options for different risk classifications are also unique. The principal treatment for clinically low-risk CaP is radical treatment, such as prostatectomy and radiotherapy. In addition to radical treatment, comprehensive treatment such as radiotherapy, chemotherapy, and endocrine therapy is required for patients with high-risk CaP. Therefore, CaP early diagnosis and grading are crucial to initiate proper treatment, improve patients' outcomes, and prolong survival.

Currently, prostate-specific antigen (PSA) is the most used diagnostic biomarker for CaP diagnosis in the clinic. The widespread use of PSA-testing has increased the diagnostic rate of CaP, but it is also accompanied by a high false-positive rate of CaP and overtreatment of indolent tumors due to the poor accuracy^[6]^. PSA cannot differentiate among benign prostate changes, indolent cancers (unlikely to cause significant symptoms), and early versus advanced stages of CaP. It often leads to 20% to 42% over-diagnosis and over-treatment, which may cause a patient more harmful than good^[7]^. The Prostate Cancer Prevention Trial showed that 14.9% of prostate tumors in men with PSA levels lower than 4.0 ng/mL had Gleason scores of seven or higher^[8]^.

Also, due to the limitations of its low specific and low sensitive character, early detection and real-time monitoring of tumor progression cannot be achieved^[9]^. Needle biopsy is the gold standard for CaP diagnosis. However, tumor biopsy has several limitations in clinical application, including the pain associated with the invasiveness of the procedure, the significant risk of hemorrhage and urinary retention, and the risk of false-negative results due to tumor heterogeneity. Magnetic resonance imaging (MRI) has somewhat improved selection for biopsy but the utility is still limited by occasional false negatives in 5%-15% of Prostate Imaging-Reporting And Data System (PIRADS) and false positives in 40%-60% of PIRADS scores 3-5 (equivocal or positive MRIs)^[10]^. There is thus an unmet need to develop tools to noninvasively detect CaP at an early stage with high sensitivity and specificity and to improve individualized treatment.

Liquid biopsy refers to the technology that makes full use of body fluids minimally or non-invasively obtained, such as blood, urine, or saliva, and it is receiving great attention as a novel diagnostic tool to access response to clinically and biologically relevant information^[11]^. EV-based liquid biopsy can be integrated to maximize insights into tumor status especially in view of dissecting tumor heterogeneity. Liquid biopsy components include circulating tumor cell, circulating tumor DNA, and/or extracellular vesicles (EVs)^[12,13]^. Compared with other types of liquid biopsy, the use of EVs such as exosomes may offer unique advantages. Firstly, exosomes are highly abundant in most biological fluids, such as blood plasma, where one can detect 10^8-13^exosome particles/mL^[14]^. Secondly, tumor-derived exosomes have specific biomarkers distinguished from exosomes from normal tissues, which can be used for cancer diagnosis and prognosis^[15]^. Furthermore, exosomes have strong stability, which means they can be stored at -80 °C for several months or even years^[16]^. Thus, exosomes are emerging as a newly attractive biomarker of liquid biopsy for non-invasive cancer diagnosis. EVs are potent and clinically valuable tools for CaP early diagnosis and prognosis as they are highly representative of their cell of origin^[17]^.

It was reported that the differential expression of EV contents in nucleic acids (i.e., DNA, mRNA, and non-coding RNAs), proteins, and lipids may create favorable conditions for CaP invasion and metastasis^[18-22]^. We have recently summarized the emerging role of EVs in liquid biopsy for monitoring CaP invasion and metastasis^[23]^. Current high-throughput technologies for genomic, transcriptomic, and proteomic analysis are driving ground-breaking discoveries in the field of new EV biomarkers. While most of the current CaP EV studies have been focused on the mechanism of metastasis and progression and as well as between cell-cell communications^[24,25]^, only a few reliable EV biomarkers for the risk classification of CaP were clinically applied.

In this commentary, we have to emphasize a very important milestone study, highlighting the clinical importance of EV biomarkers in CaP stratification, which is the discovery of a novel urine EV gene expression assay [the ExoDx Prostate IntelliScore (EPI) urine exosome assay] to differentiate high-grade CaP from low-grade CaP and avoid unnecessary biopsies^[26]^. This test has been approved by the United States Food and Drug Administration with the “Breakthrough Device” designation. The EPI test is a urine EV gene expression assay that does not require pre-collection digital rectal exam and relies on the isolation and analysis of urinary EVs. In the EPI test, first catch urine samples (25-50 mL) are collected and EVs are isolated by a proprietary ultrafiltration centrifugation technique. After extracting exosomal RNAs, the RNA copy numbers from three genes (i.e., ERG, PCA3, and SPDEF) are determined by RT-qPCR. EPI is a noninvasive, easy-to-use, urine EV-RNA assay that has been validated across three independent prospective multicenter clinical trials with 1212 subjects^[27]^. The test can discriminate high-grade (≥ GG2) from low-grade (GG1) cancer and benign disease. EPI effectively guides the biopsy-decision process independent of PSA and other standard-of-care factors. The absence of clinical variables in the EPI algorithm represents an important differentiator from other assays predicting high-grade CaP, including 4K score test (OPKO Diagnostics, Miami, FL) and SelectMDX (MDx Health, Irvine, CA). As EPI performance is based on gene expression only, this assay is more accurate than existing risk assessment methods such as clinical features. There is an option for the urologist to introduce other parameters, such as obesity status, underlying genetics, and race, for developing a more personalized risk assignment at both initial and or repeat biopsy time-points. Among urologists, 68% reported that the EPI test influenced their biopsy decision with respect to selecting the right patients to biopsy at the right time, thereby improving their ability to identify clinically significant disease and reduce biopsies when the test was negative^[28]^. Although the EPI test has achieved some satisfactory results, future studies need to incorporate it in determining the use of MRI and inclusion of the EPI-risk score into an algorithm that includes the PIRADS designation and other clinical variables. More clinical trials still need to further confirm its clinical value. In addition to the EPI test, early diagnosis or efficient prognostic EV biomarkers are warranted for improving risk stratification, personalized postoperative adjuvant therapy, and prognostication of CaP patients for clinical translation in the future.

Although the analysis of EV contents is promising in the early diagnosis and progression grading of CaP, the application of the EV-based liquid biopsy in the clinic is still facing challenges. Due to the heterogenous nature of human samples and complications in isolation, it is ideal to optimize the isolation technique to obtain relatively homogeneous EV cargoes with reproducible, high-yielding and throughput, and scale-up capability. The contaminations in blood (e.g., high-abundance blood proteins and apolipoproteins) and urine (e.g., mucoprotein, also called as the Tamm-Horsfall protein) need to be removed before genomic and proteomic analysis.

EVs act as cellular messengers and have been shown to transfer proteins and nucleic acids between tumor cells that influence tumor initiation, proliferation, progression, and metastasis^[29-31]^. An increasing number of studies have screened candidate biomarkers from body fluids that may be used to diagnose CaP. Due to the heterogeneity and variety of genetic backgrounds of patients, the lack of data from large‐scale samples for the validation of CaP biomarkers is the main deterrent to translating the potential EV biomarkers from bench to the bedside. In order to discover specific and sensitive biomarkers of tumors in diagnosis and screening, more multidisciplinary technologies and collaborations are highly expected in the near future.

The advances in high-throughput and modern omics technologies such as genomics, transcriptomics and proteomics have greatly promoted EV biomarker research in the recent decade. Machine learning has been applied to integrate multi-omics sequencing data, which are still generated at ever-growing rates and scales. Machine learning can lead to a high-quality performance for liquid biopsy-based diagnosis for multiple human cancers^[15]^ and holds promise for EV-based CaP liquid biopsy. Comprehensive multi-omics data analysis with machine learning has been a frontier in cancer genomics^[32]^ and should be performed in CaP EV biomarker research in the future [Figure 1].

**Figure 1:**
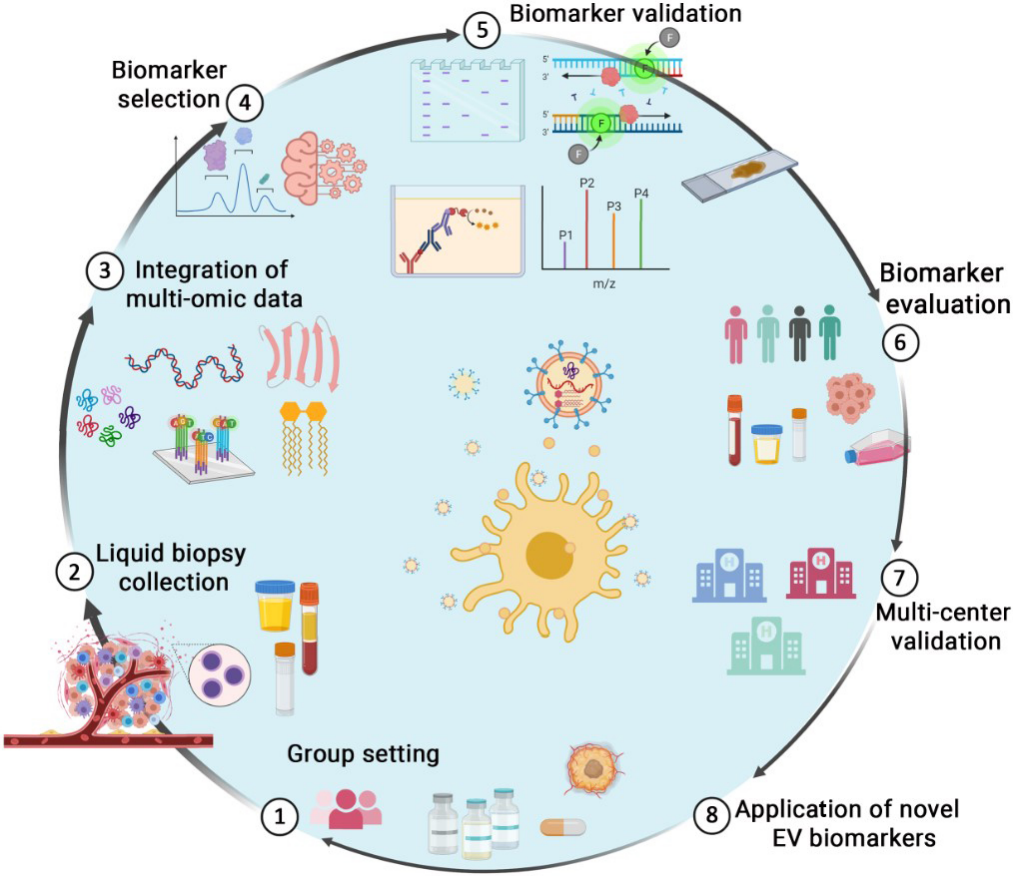
Overview of EV biomarker discovery and validation for CaP personalized treatment decision. Step 1. Group different stages of CaP patients and healthy control subjects. Step 2. Collect human samples including blood, urine, and semen. Step 3. Integrate multi-omic technology including genomics, transcriptomics, proteomics and lipidomics for profiling. Step 4. Select candidate biomarkers by machine learning and establish machine learning models. Step 5. Validate selected biomarkers by different methods such as WB, qRT-PCR, ELISA, PRM, and immunofluorescence in samples such as blood, urine, tissue, and cultured cell lines. Step 6. Evaluate selected biomarkers in a large set of independent patient samples. Step 7. Multi-center EV biomarker diagnosis performance verification. Step 8. Novel EV biomarkers for early diagnosis and patient grading, and for precision medicine. ELISA: Enzyme-linked immunosorbent assay; CaP: prostate cancer; EV: extracellular vesicle; qRT-PCR: quantitative reverse transcription PCR; PRM: parallel reaction monitoring; WB: western blot.

Oncogenesis is driven by a complex and intricately controlled gene expression programming related to molecular-level variation in many genes. Due to the high heterogeneity and intricacy, a single biomarker does not fully characterize tumor properties, and more accurate predictions using multi-parameter markers are required^[33,34]^. A set of candidate biomarkers can be evaluated for their differences in abundance between patients and normal controls, resulting in more robust predictive and diagnostic capacities^[35]^. For CaP EV-based biomarker research, a comprehensive pipeline for discovery and validation is shown in Figure 1.

Most importantly, these putative EV biomarkers need further validation independently at multiple levels, such as blood, urine, tumor tissue, and cell lines, to increase their specificity and sensitivity before clinical application. During this stage, the sensitivity and specificity of the selected EV markers need to be compared with the current PSA test, MRI imaging, and tissue biopsy. These markers need to ensure they are disease-specific, rather than trial-dependent. Assessing multi-center EV biomarker diagnosis performance in CaP is necessary to further evaluate the clinical value of these biomarker set prior to its widespread use. These EV biomarkers can be used to diagnose CaP and predict the stages of cancer and the tumor biological activity. This EV-based liquid biopsy can guide clinicians in choosing the best treatment methods for an individual CaP patient and significantly improve their prognosis.

## DECLARATIONS

### Copyright

© The Author(s) 2022.
